# Dietary soybean meal affects intestinal homoeostasis by altering the microbiota, morphology and inflammatory cytokine gene expression in northern snakehead

**DOI:** 10.1038/s41598-017-18430-7

**Published:** 2018-01-08

**Authors:** Shuyan Miao, Chenze Zhao, Jinyu Zhu, Juntao Hu, Xiaojing Dong, Longsheng Sun

**Affiliations:** College of Animal Science and Technology, Yangzhou University, 48 Wenhui East Road, Yangzhou, 225009 China

## Abstract

A 63-day feeding trial was conducted in northern snakehead to observe the effects of a dietary soybean meal substitution on the microbiota community, morphology and inflammatory cytokine gene expression in the intestine. Four isonitrogenous and isoenergetic diets containing increasing levels of soybean meal were used to replace 0%, 25%, 50% and 75% of the defatted fishmeal (diets are referred to G1, G2, G3 and G4, respectively). Different dietary soybean meal substitutions significantly affected the intestinal microbiota composition. At the phylum level, Firmicutes abundance was the lowest in the G4 group, in contrast with Proteobacteria, Bacteroidetes and Planctomycetes. At the genus level, significantly lower abundance of *Lactococcus*, *Geobacillus*, *Pseudomonas*, *Streptococcus*, *Bacillus* and *Acinetobacter*,but higher abundance of *Cetobacterium*, *Planctomyces*, *Shewanella*, *Thermomonas*, *Rubrivivax* and *Carnobacterium* was observed in fish fed the G4 diet. With increased dietary soybean meal, the thickness of the muscularis, the height of the fold and the height of the microvillus in the distal intestine decreased, but the relative expression of IL-1β, IL-10 and IL-17F was significantly up-regulated. In conclusion, more emphasis should be placed on the functionality of intestinal microbiota and the pathogenesis of mucosal inflammation to assess the effects of diet and fish intestinal health through intestinal microbiota profiling.

## Introduction

Fishmeal is an important protein source in aquafeeds, placing increasing pressure on feed cost and wild fish stocks^1^.Replacing fishmeal with plant-based ingredients has attracted increasing attention, due to their relative availability and competitive prices. Soybean meal has many advantages, including a high protein content^2^, a relatively balanced amino acid content and a high digestibility profile^3^; therefore, soybean meal is the most widely used substitution of fishmeal among the plant protein in aquatic animals. Yaghoubi *et al*. demonstrated that soybean meal and isolated soy protein could replace 27.3% of dietary fishmeal without negatively affecting the growth performance in juvenile silvery-black porgy; however, a higher dietary soybean content induced signs of hyperlipidemia and anemia signs^4^. Similarly, several adverse effects on growth performance^5^, digestibility of nutrients^6^, feed efficiency^7^, immune responses^8^ and intestinal health^2^ were observed. Certain indigestible components (non-starch polysaccharides)^9^, and some anti-nutritional factors and low concentrations of lysine, methionine and threonine were considered restricting the use of soybean in aquafeeds^10^.

Among all the adverse reactions to soybean product substitution in fish, the effects on the intestinal health, such as alterations in the gut histology and immunosuppression, have received significant attention^11,12^. Several studies have demonstrated that although a 205 g/kg soybean meal substitution of fishmeal produced the maximum growth in gilthead sea bream, and 300 g/kg of soybean meal level did not exert a significant adverse effect on the growth performance and feed utilization in gilthead sea bream juveniles, higher levels of soybean meal led to the presence of cellular infiltration of the submucosa and lamina propria^13^. Merrifield *et al*. fed rainbow trout with 46% soybean meal in replacement of 50% fishmeal for 16 weeks, and at the end of the study, shorter posterior and less dense anterior intestine microvilli were detected^14^. These histological changes in the intestinal tract could be related to certain functional disturbances, such as enteritis, changes in absorptive cells and increased presence of inflammatory cells^15,16^. Furthermore, previous studies have shown that soybean meal could induce immune-related gene expression in the intestine and inflammatory response changes in Atlantic salmon^17,18^.

The intestinal microbiota plays a critical role in aiding digestive function and the formation of a defensive barrier to protect the fish against pathogenic invasions^19,20^, thus, a healthy intestinal microbiota is essential for promoting fish health and well-being. Many factors, such as the species, stages and environmental factors, can modulate the intestinal microbiota community^21^. Undoubtedly, diet is an important factor affecting the intestinal microbiota^22^. However, the general knowledge regarding the correlations among fish intestinal microbiota, dietary feed and intestinal health remains incomplete and unclear. For example, in mammals, Lepage *et al*. used high-throughput sequencing techniques to demonstrate that certain human health disorders, such as inflammatory bowel disease might be closely associated with gut microbial dysbiosis^23^. The same phenomenon was also observed in mice^24^. The effects of soybean meal on the intestinal microbiota in fish have been observed in previous studies^14,25,26^, but few studies investigating the relationship between the intestinal microbiota and immune functions have been well documented.

The northern snakehead (*Channa argus* Cantor, 1842) is a member of the *Channidae* family. Recently, this fish species has become widely cultured in southern and southeastern Asian countries^27^. Its annual output was nearly 500,000 tons in 2015 in China^28^. As a carnivorous fish, the protein requirements of northern snakehead juveniles were 47.9–50.5% at a dietary lipid level of 6.5–12.0%^29^. Therefore, the bulk of fishmeal in the diet is required in diet to meet the balanced amino acids and good digestibility for good growth performance. Due to the cost of the formulated diets, substituting fishmeal with plant protein sources, i.e., soybean meal, is necessary in northern snakehead culture. The primary aims of the present study were to determine (1) the effects of dietary soybean meal on the intestinal microbiota innorthern snakehead, (2) the intestinal health, including the intestinal morphology and expression of inflammatory cytokines in response to dietary soybean meal and (3) the relevance of diet, fish intestinal microbiota and intestinal health.

## Results

### Survival rate and growth performance

The effects of different dietary soybean meal levels on the survival rate and growth performance of the fish are presented in Table 1. No difference was observed in the survival rate among different groups (*P* > 0.05). However, the different soybean meal substitutions significantly affected the growth performance (*P* < 0.05). The fish in the G1 and G2 groups showed a higher WGR and SGR than the fish in the G3 and G4 groups, while the WGR and SGR were the lowest in the fish fed the G4 diet (*P* < 0.05). However, no significant difference was observed in the WGR and SGR between the fish in the G1 group and those in the G2 group (*P* > 0.05).

**Table 1:** Survival and growth performance of northern snakehead in the different groups (mean ± S.D. of three replications).

Groups	Initial body weight (W_0_, g)	Final body weight (Wt, g)	Weight gain rate (WGR, %)	Specific growth rate (SGR, %)	Survival rate (%)
G1	8.68 ± 0.09	40.35 ± 2.52^a^	364.86 ± 17.86^a^	2.44 ± 0.16^a^	88.00 ± 2.30^a^
G2	8.58 ± 0.06	38.75 ± 3.28^ab^	351.44 ± 16.99^a^	2.39 ± 0.10^a^	86.67 ± 1.33^a^
G3	8.61 ± 0.12	33.59 ± 2.72^b^	290.08 ± 13.90^b^	2.16 ± 0.09^b^	86.67 ± 4.62^a^
G4	8.66 ± 0.12	25.25 ± 2.43^c^	191.77 ± 10.89^c^	1.70 ± 0.11^c^	88.00 ± 2.30^a^

Means shown on the same line with different superscript letters are significantly different (*P* < 0.05) according to Tukey’s test.

### Intestinal microbiota composition

#### Classification and Alpha diversity analysis

The OTUs and Alpha diversity statistics of the intestinal microbiota in northern snakehead are presented in Table 2. No significant differences in the OTUs were observed in the intestines of fish fed the G1, G2 and G3 diets (*P* > 0.05), and the lowest OTUs were observed in the G4 group (*P* < 0.05). The Sobs, Chao and ACE indices in the G4 group were significantly higher than those in the G1, G2 and G3 groups (*P* < 0.05), in contrast to the Simpson index (*P* < 0.05). Meanwhile, no significant difference in the Sobs, Chao, ACE and the Simpson indices were observed among the fish fed the G1, G2 and G3 diets (*P* > 0.05). The Shannon index was higher in the fish fed the G2 and G4 diets than those fed the G1 and G3 diets (*P* < 0.05).

**Table 2:** OTUs and Alpha diversity statistics of the microbial sequencing of the northern snakehead intestine (mean ± S.D. of three replications).

**Groups**	**Reads**		**Tags**	**OTUs**	**97% similarity**				
	**Raw reads**	**Valid reads**			**Sobs**	**Chao**	**ACE**	**Shannon**	**Simpson**
G1	49764	42297	40734	157 ± 12^a^	157 ± 9^a^	163 ± 11^a^	168 ± 10^a^	2.16 ± 0.11^a^	0.22 ± 0.01^b^
G2	43826	41837	41701	165 ± 17^a^	165 ± 13^a^	165 ± 12^a^	167 ± 9^a^	2.40 ± 0.09^b^	0.25 ± 0.02^b^
G3	47084	41939	41149	150 ± 16^a^	150 ± 11^a^	159 ± 9^a^	164 ± 12^a^	2.17 ± 0.13^a^	0.22 ± 0.00^b^
G4	43414	41385	41193	230 ± 19^b^	230 ± 14^b^	231 ± 21^b^	233 ± 17^b^	2.48 ± 0.16^b^	0.18 ± 0.02^a^

Means shown in the same column with different superscript letters are significantly different (*P* < 0.05) according to Tukey’s test.

### Microbiota composition and relative abundance analysis

**Figure 1:**
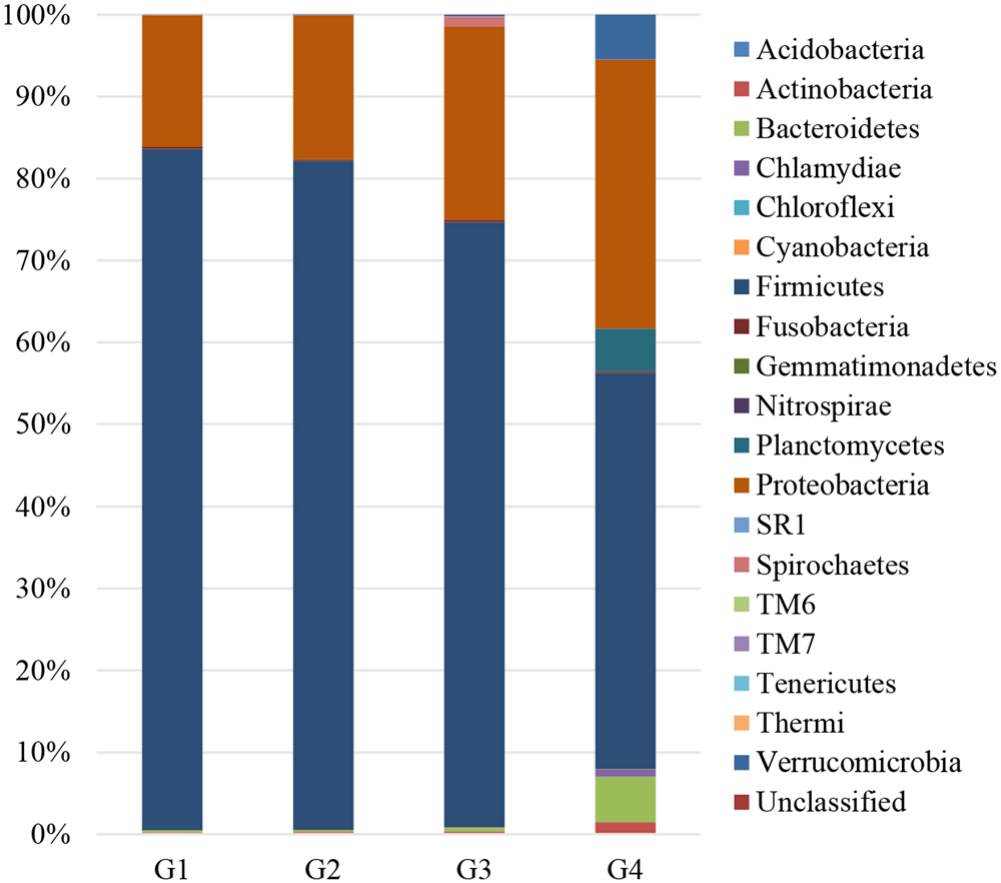
The average relative abundance at the phylum level in the intestine of northern snakehead.

The microbiota composition at the phylum level in the intestine of northern snakehead is represented in Fig. 1. In total, 19 phyla were detected in the intestines of the fish fed different diets. Firmicutes (48.29–83.15%) and Proteobacteria (16.10–32.81%) were the most abundant phyla, and no significant difference in the abundance of Firmicutes and Proteobacteria was observed among the G1, G2 and G3 groups (*P* > 0.05). However, the abundance of Firmicutes was significantly higher in the G1, G2 and G3 groups than that in the G4 group, in contrast to the abundances of Proteobacteria and Bacteroidetes (*P* < 0.05). Moreover, Planctomycetes were only detected in the intestines of fish fed the G4 diets, with an abundance of 5.23%.

**Figure 2:**
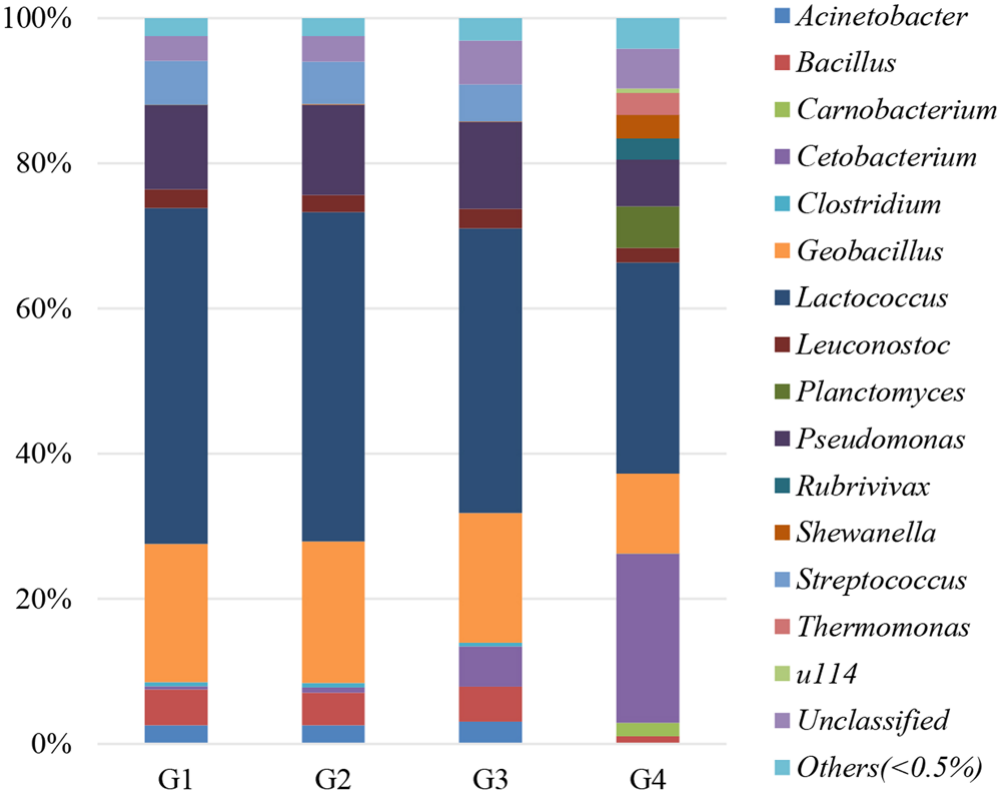
The average relative abundance at the genus level in the intestine of northern snakehead.

The microbiota composition at the genus level in the intestine of northern snakehead is represented in Fig. 2. Regardless of the type of diet, *Lactococcus* (29.09–46.24%) was the most abundant genus in the intestines of the fish. Compared to the genus abundance in the intestines of the fish in the G1, G2, and G3 groups, a significantly lower abundance of *Lactococcus*, *Geobacillus*, *Pseudomonas*, *Streptococcus*, *Bacillus* and *Acinetobacter* was detected in the fish fed the 4 diet (*P* < 0.05). However, the abundance of *Cetobacterium*, *Planctomyces*,* Shewanella*, *Thermomonas*, *Rubrivivax* and *Carnobacterium* was the highest in the intestines of fish fed the G4 diet (*P* < 0.05). Meanwhile, *Carnobacterium*, *Planctomyces* and *Thermomonas* were not detected in the other three groups. Furthermore, the abundance of *Cetobacterium* was the highest in the fish fed the G4 diet, followed by the fish fed the G3 diet, and the lowest abundance was observed in the fish fed the G1 and G2 diets (*P* < 0.05).

### Distal intestinal morphology

The changes in TM, HF and HMV in the distal intestines of the fish were caused by the different diets. As indicated in Table 3, the TM of the fish in the G4 group was 88.74 μm, which was significantly lower than that in the fish fed the G2 diet (*P* < 0.05). The HF in the G3 and G4 groups was significantly lower than that in the G1 group (*P* < 0.05). Meanwhile, no significant difference in HF was observed between the fish in the G2 group and those in the other three groups (*P* > 0.05). The HMV of the fish in the G2 group was the highest, followed by the fish in the G1 and G3 groups. However, the G4 diet resulted in the lowest HMV in the distal intestine (*P* < 0.05).

**Table 3:** Distal intestine morphology of northern snakehead in the different groups (mean ± S.D. of three replications).

**Groups**	**TM (μm)**	**HF (μm)**	**HMV (μm)**
G1	110.21 ± 10.67^a^	295.32 ± 15.61^a^	25.59 ± 1.79^a^
G2	114.53 ± 9.78^a^	268.69 ± 12.31^ab^	28.92 ± 2.68^a^
G3	104.52 ± 10.10^ab^	254.59 ± 19.17^b^	20.16 ± 2.00^b^
G4	88.74 ± 9.23^b^	242.34 ± 19.95^b^	15.28 ± 1.47^c^

Means shown in the same column with different superscript letters are significantly different (*P* < 0.05) according to Tukey’s test.

TM: thickness of the muscularis; HF: height of the fold; HMV: height of the microvillus.

### Relative expression of inflammatory cytokine genes

**Figure 3:**
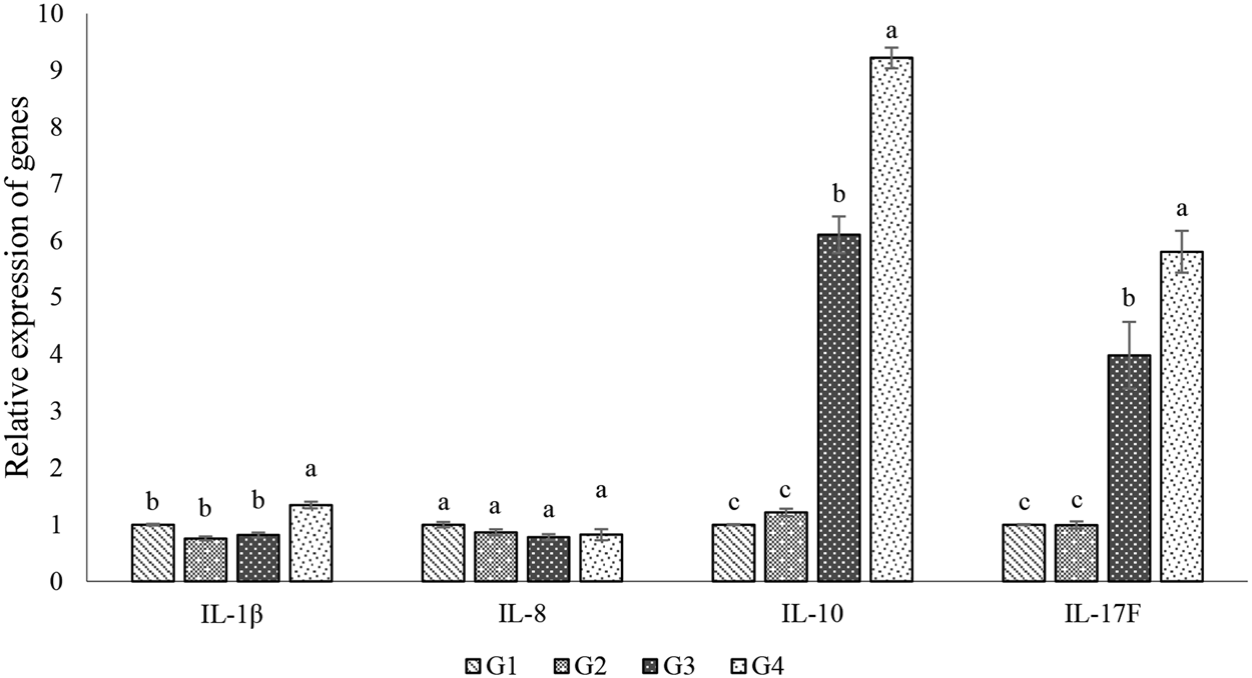
Relative expression of immune-related genes in the intestine of northern snakehead. Relative expression of immune-related genes was presented as mean ± S.D. (n = 3).

The effects of the soybean meal substitutions on the gene expression of inflammatory cytokines in the intestine of northern snakehead are shown in Fig. 3. After 63 days, no changes in the expression of IL-8 were observed in the intestines of the fish fed the different dietary levels of soybean meal (*P* > 0.05). The relative expression of IL-1β was not significantly different in the intestines of the fish fed the G1, G2 and G3 diets (*P* > 0.05) but was significantly up-regulated in the G4 group (*P* < 0.05). The relative expression of IL-10 and IL-17F was significantly up-regulated in the G3 and G4 groups compared to that in the fish in the G1 and G2 groups (*P* < 0.05). In addition, the relative expression of IL-10 and IL-17F was the highest in the fish fed the G4 diet (*P* < 0.05).

## Discussion

At the level of the soybean meal substitution increased, the TM, HF and HMV of the distal intestine in northern snakehead exhibited a decreasing trend in the present study, which is consistent with studies investigating Japanese flounder^30^ and Japanese seabass^31^. Because the distal intestine is highly sensitive to dietary anti-nutritional factors, morphological studies of this organ have been conducted^31,32^. A disruption in the mucosal integrity, reduction in the mucosal fold, infiltration of inflammatory cells and abnormal vacuolization, which may lead to negative effects on the growth and health of the fish, are often observed in the intestine^33,34^. The anti-nutritional factors in SBM, such as saponin^30^ and lectin^35^, were considered the main causes of the changes in the intestinal morphological parameters. Moreover, Knudsen *et al*. showed that soybean saponins were resistant to degradation in the gut of Atlantic salmon^36^, resulting in the accumulation of these compounds in the distal intestine^37^, and the excessive defoliation of the intestinal mucosa^38^. Chen *et al*. showed that 0.8 g kg^−1^ saponin did not change the distal intestinal histological structure in the Japanese flounder. However, increasing dietary saponin to 3.2 and 6.4 g kg^−1^ significantly impaired the intestinal villi and goblet epithelium^30^. Furthermore, soybean saponins alter the membrane permeability and disrupt the intestinal cell membranes^39–41^. Additionally, the lectins in soybean meal could combine with polysaccharides on the intestinal epithelial cell surface and impair the gut microvilli^42^.

Many other factors, such as unbalanced amino acids^43^, soybean proteinase inhibitors^44^ and β-conglycinin^45^, could also exert an adverse impact on the absorption and nutrient digestion in the intestine, further resulting in poor growth performance in fish fed higher dietary soybean meal levels. In the present study, a 22% dietary soybean meal, i.e., the 25% substitution of the fishmeal protein, did not affect the growth performance of northern snakehead compared to the fishmeal diet. However, the effect of soybean meal on growth was substitution-related, and the growth performance became increasingly compromised as the soybean meal substitution levels increased, which is consistent with previous studies^6,46–48^, particularly in diets in which soybean meal was the main protein source^49^. Nevertheless, Laporte and Trushenski showed that sunshine bass could tolerate a high soybean meal content in terms of the balance of the intestinal function and integrity^50^. Webster *et al*.^51,52^ and Kaushik *et al*.^53^ reported that the growth performance of juvenile blue catfish, channel catfish and rainbow trout was unaffected by the complete substitution of fishmeal with soybean meal and soy protein concentrate. This discrepancy might be due to the fish species and stages, living conditions and diets^33^.

The intestinal microbiota, particularly its effect on gut health, in aquatic animals has gained increasing attention^14,25,31,54^. Regardless of the type of diet, Firmicutes and Proteobacteria were the most dominant in the intestines of northern snakehead in all groups in the present study. At the genus level, *Lactococcus*, *Geobacillus* and *Pseudomonas* were the most dominant genera. In studies on investigating protein source substitutions, increasing emphasis was placed on the changes of the intestinal microbiota induced by different protein sources. A 75% soybean meal substitution with fishmeal protein exerted an adverse effect on the relative abundance of Firmicutes, particularly on certain genera, including *Lactococcus*, *Geobacillus*, *Streptococcus*, *Bacillus*and *Acinetobacter*. Except for *Acinetobacter*, the other genera belong to beneficial microorganisms, such as lactic acid bacteria, that often settle on the intestinal epithelium and form a barrier against various microbial pathogens^19,55^. However, Gajardo *et al*. reported that the relative abundance of lactic acid bacteria was 18 times higher in the intestines of Atlantic salmon fed soybean meal than that in fishmeal-fed fish, and the higher level of indigestible fiber present in soybean meal was presumably as the primary cause because lactic acid bacteria utilize such substrates for their metabolism and growth^54^. Moreover, the abundance of Proteobacteria (including *Shewanella*, *Thermomonas*, and *Rubrivivax*), Planctomycetes (including Planctomyces) and Bacteroidetes (including *Cetobacterium*) were all increased in the fish fed the G4 diet. Certain species of *Shewanella*, *Carnobacterium*, *Thermomonas and Planctomyces* are opportunistic pathogens that could impair the intestinal immune mechanisms in fish^56^. Considering these findings, the results of the present study are worthy of further investigation prior to the utilization of soybean meal in northern snakehead. In addition, the changes in the intestinal microbiota composition, including the dominant phyla and genus were soybean meal substitution-related^31^, which was also consistent with the results of the current study.

In contrast, the quantitative effects of the soybean meal substitution on the intestinal microbiota were marginal, but the tentative taxonomic characterization of all microorganisms revealed some observable differences, particularly in the fish fed the highest content of soybean meal, which was consistent with observations in juvenile rainbow trout^17^. The Alpha diversity statistics of microbial sequencing further verified this result. However, this finding was inconsistent with the report in allogynogenetic silver crucian carp, which demonstrated no changes in the diversity and richness indices of a microbial community following the addition of 30% soybean meal to replace 59% of fishmeal protein^57^. This discrepancy could be due to the following: exogenous and endogenous factors, such as living conditions, feed composition, fish species and stage, and fish intestinal morphology^58^. For example, the host phylogenetic position and living environment might be the most important determinants of the diversity of intestinal microbiota in Japanese seabass^31^.

The constant exposure to the water environment generates the mucosal epithelia of fish matter, reflecting its defense-barrier function against physical,biological and chemical hazards^59^. Therefore, mucosal immunity has become a new hotspot in immunology studies. The important role played by the intestinal microbiota in the modulation of the mucosal immunity response, particularly its function in the pathogenesis of inflammatory bowel disease has been confirmed in humans and animals^60,61^. For example, Crohn’s disease patients treated with a sterile effluent ultra filtrate did not show triggered inflammation, whereas the reintroduction of small bowel effluent resulted in inflammation^62^. One mechanism of the microbiota involved in regulating mucosal immunity has been discussed by Rakoof-Nahoum *et al*., who reported that Toll-like receptors (TLRs) could recognize commensal bacteria under normal conditions, and those interactions between microbial pattern recognition receptors and commensal bacterial products played a major role in intestinal homeostasis and the resistance to epithelial injury. Thus, a dysregulated interaction between bacteria and TLRs may promote chronic inflammation^63^. In addition, Nayak reported that *Bacillus* could decrease inflammation via the up-regulated secretion of anti-inflammatory cytokines^64^. Proteobacteria and Bacteroidetes, including certain opportunistic pathogens, showed a higher relative abundance in the fish in the G4 group, which should be further explored in follow-up experiments to clarify the causes and impact on health.

The mucosal immune system in fish includes certain immunocompetent cells and factors in the intestinal mucous membrane. Of these factors, the interleukins (ILs), interferon regulatory factors (IRFs) and tumor necrosis factors (TNFs) are the main immune-relevant factors linked to inflammation in the distal intestine in fish^18^. Because the excessive dietary plant protein sources typically lead to intestinal inflammation^18,65^, the gene expression of certain inflammatory cytokines (IL-1β, IL-8, IL-10 and IL-17F) was measured to test the effect of soybean meal on the mucosal immune system in northern snakehead in the present study. Consistent with the observations reported in previous studies^54,66^, soybean meal affected the gene expression of certain factors. The up-regulated relative expression of IL-1β in the fish fed the G4 diet was consistent with the observations in Atlantic salmon. However, the level of IL-1β observed in G4 was only 1.6-fold higher than that in G1 after the 63-d trial, while that observed in Atlantic salmon was 20-fold higher^18^. The effect of dietary soybean meal on the expression of IL-1β reflect the fish species and stages due to the different tolerance capability for soybean meal^18^.The substantial up-regulation of the relative expression of IL-17F in the fish fed the G3 and G4 diets deserves further investigation. As pro-inflammatory cytokines, IL-1β and IL-17F have been implicated in the disease pathogenesis^67,68^, such as inflammatory bowel disease and celiac disease in humans^69,70^. As an anti-inflammatory cytokine, the relative expression of IL-10 in the fish fed the G3 and G4 diets was also up-regulated; the same trend was observed in carp intestine during early disease stages, whereas a down-regulated expression was observed in Atlantic salmon during the late stages^18,71^. The different responses of IL-10 during different stages of inflammation indicate that IL-10 might play a major part in the recovery from inflammation. To prevent the related damages resulting from inflammation, suppression of the early inflammatory processes during long-term inflammation was observed in mammals as a protective mechanism^72,73^. However, the gradual changes in factors during inflammation progression and other factors related to inflammation should be observed to clarify the regulatory mechanism of cytokines in the pathogenesis of inflammation.

In conclusion, the present study confirms that dietary soybean meal significantly affects the diversity and composition of intestinal microbiota. The fish fed the higher levels of dietary soybean meal had a lower relative abundance of lactic acid bacteria but a higher abundance of opportunistic pathogens. In addition, the changes in TM, HF and HMV in the distal intestine of these fish reflected the higher dietary levels of soybean meal. The relative expression of IL-1β, IL-10 and IL-17F was significantly up-regulated. Moreover, these effects are partially related to the soybean meal substitution. The link between dietary soybean meal and fish intestinal microbiota and the related changes in intestine health require further investigation.

## Materials and Methods

### Ethical statement

The animal study proposal was approved by the Institutional Animal Care and Use Committee (IACUC) of the Yangzhou University Animal Experiments Ethics Committee (permit number: SYXK (Su) IACUC 2012-0029). All experimental procedures were performed in accordance with the Regulations for the Administration of Affairs Concerning Experimental Animals approved by the State Council of the People’s Republic of China.

### Experimental diets

The dietary ingredients and the proximate composition of the isonitrogenous and isoenergetic diets are shown in Table 4. The main protein source in the control diet was defatted fishmeal and the other three diets contained increasing contents of soybean meal to substitute 25%, 50% and 75% of the fishmeal. The four diets were referred to G1, G2, G3 and G4. All ingredients were ground into a fine powder using a mini-type mill (HK820, Guangzhou Xulang Machinery Manufacturing CO., LTD, China) with a 246-μm mesh. Subsequently, the material powder and fish oil were thoroughly mixed and distilled water was gradually added to create a stiff dough for processing the experimental diets using a feed mill (F-26Ш, South China University of Technology, Guangzhou, China). The pellets were dried at 50 °C, cut into approximately 2.0 × 3.0 mm sections, and subsequently stored at −20 °C.

**Table 4:** Ingredients and compositions of the experimental diets (%, dry matter basis).

**Ingredients**	**Content (g/100 g diet)**			
	**G1**	**G2**	**G3**	**G4**
Defatted fishmeal	60.00	45.00	30.00	15.00
Soybean meal	0.00	22.00	47.00	70.00
Wheat flour	19.30	19.30	12.44	4.44
Wheat bran	10.14	3.14	0.00	0.00
Fish oil	4.00	4.00	4.00	4.00
Vitamin premix^a^	0.50	0.50	0.50	0.50
Mineral premix^a^	0.50	0.50	0.50	0.50
Calcium dihydrogen phosphate	1.50	1.50	1.50	1.50
Choline chlorine (95%)	1.00	1.00	1.00	1.00
Soybean lecithin	2.00	2.00	2.00	2.00
Sodium alginate	1.00	1.00	1.00	1.00
Ethoxyquin	0.03	0.03	0.03	0.03
Vitamin C	0.03	0.03	0.03	0.03
Proximate analysis (%)				
Crude protein	45.31	45.50	45.15	44.97
Crude lipid	9.15	9.10	9.07	9.17
Ash	12.87	12.85	12.75	12.79
NFE^b^	32.67	32.55	33.03	33.07
Gross energy (kJ/g)^c^	19.93	19.93	19.92	19.93

^a^Kindly provided by Qingdao Master Bio-Tech Co. Ltd. (Qingdao, Shandong, China).

^b^Nitrogen free extracts (NFE) = dry matter - (crude lipid + crude ash + crude protein).

^c^Gross energy was calculated using factors of 23.64, 39.54 and 17.15 kJ g^−1^ for protein, lipid and carbohydrate, respectively^43^.

### Fish and experimental conditions

The trial was performed in a water-recycling system at Yangzhou University, Jiangsu, China. Northern snakehead juveniles were obtained from a commercial farm (Gaoyou, Jiangsu, China). A total of 600 juveniles were cultured in three 4 m^2^ cement ponds at about 26.5–28.0 °C for 2 weeks to allow them to acclimate to the water condition. During the acclimation period, 1/3 of the water was changed daily, and the juveniles were fed by hand with a commercial diet (Haid Group, Guangzhou, China). Prior to the trial, the fish were not fed for 24 h, and subsequently 300 healthy fish (8.65 ± 0.25 g) were randomly distributed into 12 tanks (350 L) in a water-recycling system. Each tank held 25 fish, and each diet had three replicate tanks. The fish were fed twice daily at 08:00 and 17:00 to an apparent satiation level. The fish mortalities were recorded daily, and certain indicators of rearing water were determined every seven days. The temperature was27.0 ± 1.5 °C, the pH was 7.23 ± 0.15, and the dissolved oxygen concentration was above 5.5 mg/L. The total ammonia nitrogen level was below 0.14 mg/L, and the level of nitrite-N was below 0.08 mg/L.

### Sample collection

After the 63-day trial, the fish were not fed for 24 h. Subsequently, MS-222 (250 mg/L, Sigma) was added into the tanks to relieve stress prior to sampling. All fish in each tank were weighed and counted, and the weight gain rate (WGR), specific growth rate (SGR) and survival rate (SR) were calculated.

To analyze the intestinal microbiota, three fish from each tank were randomly sampled and subsequently dissected under aseptic conditions to obtain the intestines. All samples from fish fed the same diet were gathered into one Eppendorf tube and subsequently immediately stored in liquid nitrogen until DNA extraction. To analyze the immune factors, the intestines from 8 fish per tank were cut into 1.5–2.0 cm segments, placed in RNAlater (Qiagen, Germany) at 4 °C overnight and subsequently stored at −20 °C. To observe the intestinal morphology, 3 fish were randomly selected from each tank, the intestine was removed, and 5 mm-long segments were sampled from the distal intestine, fixed in Bouin’s fixative solution for 24 h, and gradually transferred into 70% ethanol (ethanol/water, v/v) for 24 h.

### Sample analysis

#### Proximate composition analysis of the feed ingredients and experimental diets

The proximate composition of the ingredients and diets was determined using the methods proposed by the AOAC^74^. Crude protein was measured using the Kjeldahl method and a Kjeltec Auto Analyzer (8400, FOSS Analytical AB, Hoganas, Sweden). Crude lipid was measured using the Soxhlet method. Ash was measured using the combustion method at 550 °C for 24 h.

### Intestinal microbiota community detection

The 16 S sequencing of the intestine microbiota community was performed according to Miao *et al*.^75^. Briefly, total bacterial DNA was isolated from the intestinal samples using the QIAamp^®^ DNA Stool Mini Kit (QIAGEN, cat#51504), and for microbial detection, the V3-V4 regions of the 16 S rDNA were amplified using PCR. High-throughput sequencing was performed using an Illumina MiSeq PE250 sequencer. The high-throughput sequencing data were processed using FLASH v1.2.11, and subsequently operational taxonomic units (OTUs) were produced using USEARCH GLOBAL at 97% similarity. Finally, the OTUs in each sample were assigned using the Ribosomal Database Project (RDP) Classifier v.2.2.

### RNA extraction and qRT-PCR

The frozen intestinal tissue was ground into a fine powder in liquid nitrogen with a pre-chilled mortar and pestle. Five milligrams of ground tissue was dissolved directly in 1 mL TRIzol reagent (Invitrogen, Carlsbad, CA, USA) for total RNA extraction according to the manufacturer’s instructions. After extraction with chloroform, precipitation with isopropanol and washings with 70% (vol/vol) ethanol, and extracted RNA was resuspended in RNase-Free Water. RNA integrity and quality were assessed using the Nano Drop 1000 spectrophotometer (Thermo Scientific, Wilmington, DE, USA) and Agilent Bioanalyzer 2100 (Agilent technologies, Santa Clara, CA, USA), respectively. The RNA was treated with gDNA Eraser (Takara, Japan), and 1.0 μg was used for reverse transcription with a PrimeScript RT Reagent Kit (Takara, Japan).

Specific primers for IL-1β, IL-8, IL-10, IL-17F and β-actin were designed using Primer Express software (Version 3.0, Applied Biosystems, CA, USA). The primer sequences are listed in Table 5 and synthesized by Sangon (Shanghai, China). The qPCR primers quality were assessed by amplification efficiency and melting curve. ß-actin was chosen as reference gene from certain reference genes (such as 18 s rRNA, ß-actin, GAPDH, and EF1-α) based on preliminary tests using geNorm (version 3.5) and NormFinder algorithms^76,77^.

**Table 5:** Primers used for the real-time PCR (RT-PCR) analysis.

**Primer name**	**Primer sequence (Forward**, **5′ to 3′)**	**Primer sequence (Reverse**, **5′ to 3′)**
IL-1β	GTTTACCTGAACATGTCGGC	AGGGTGCTGATGTTCAGCCC
IL-8	CTATTGTGGTGTTCCTGA	TCTTCACCCAGGGAGCTTC
IL-10	CAGTGCAGAAGAGTCGACTGCAAG	CGCTTGAGATCCTGAAATATA
IL-17F	GTCTCTGTCACCGTGGAC	TGGGCCTCACACAGGTACA
β**-**actin	TTGAGCAGGAGATGGGAACCG	AGAGCCTCAGGGCAACGGAAA

Real-time quantitative reverse transcriptase-polymerase chain reaction (qRT-PCR) analysis was performed by SYBR Green Master Mix (Takara, Japan) with a 7500 Real-Time PCR System (Applied Biosystems, USA) to assay the relatively quantitative mRNA expression of IL-1β, IL-8, IL-10 and IL-17F in intestine of northern snakehead. The analyses of the data for relative gene expression were performed using the 2 ^−△△CT^ method^78^.

### Intestinal morphology

The distal intestine samples were stained with hematoxylin and eosin (H&E). Subsequently, the intestinal morphology, including the thickness of the muscularis (TM), the height of the fold (HF) and the height of the microvillus (HMV), was observed under a light microscope (OLYMPUS, DP73) according to Wang *et al*.^33^.

### Calculations and statistical methods

The SR, WGR and SGR were calculated using the following formulas, and the WGR and SGR calculations were based on the mean overall weight of each tank.

$$\begin{array}{rcl}{\rm{SR}}( \% ) & = & 100\times ({\rm{Nt}}/{{\rm{N}}}_{0}),\\ {\rm{WGR}}( \% ) & = & 100\times ({\rm{Wt}}-{{\rm{W}}}_{0})/{{\rm{W}}}_{0},\\ {\rm{SGR}}( \% ) & = & 100\times (\mathrm{ln}\,\overline{{\rm{W}}}{\rm{t}}-\,\mathrm{ln}\,{\overline{{\rm{W}}}}_{0})/{\rm{t}};\end{array}$$

where Nt, N_0_, W_t_, W_0_, and t represented the final number of fish, the initial number of fish, the final fish body weight, the initial fish body weight and the days of the experimental period, respectively.

All statistical analyses were conducted using SPSS 20.0 (SPSS Inc., Michigan Avenue, Chicago, IL, USA) for Windows. All results are expressed as the means ± S.D. All data were subjected to one-way ANOVA, and Tukey’s test was performed to compare the mean values between individual treatments.
